# Cannabinoids, cannabis, and cannabis-based medicine for pain management: a protocol for an overview of systematic reviews and a systematic review of randomised controlled trials

**DOI:** 10.1097/PR9.0000000000000741

**Published:** 2019-04-30

**Authors:** Emma Fisher, Christopher Eccleston, Louisa Degenhardt, David P. Finn, Nanna B. Finnerup, Ian Gilron, Simon Haroutounian, Elliot Krane, Andrew S.C. Rice, Michael Rowbotham, Mark Wallace, R. Andrew Moore

**Affiliations:** a Centre for Pain Research, University of Bath, Bath, United Kingdom; b Cochrane Pain, Palliative, and Supportive Care Review Groups, Oxford University Hospitals, Oxford, United Kingdom; c Department of Clinical and Health Psychology, Ghent University, Ghent, Belgium; d National Drug and Alcohol Research Centre, University of New South Wales, Sydney, Australia; e Pharmacology and Therapeutics, School of Medicine, Galway Neuroscience Centre and Centre for Pain Research, NCBES, National University of Ireland Galway, Galway, Ireland; f Department of Clinical Medicine, Danish Pain Research Center, Aarhus University, Aarhus, Denmark; g Department of Neurology, Aarhus University Hospital, Aarhus, Denmark; h Department of Anesthesiology and Perioperative Medicine, Kingston General Hospital, Queen's University, Kingston, ON, Canada; i Centre for Neuroscience Studies, Queen's University, Kingston, ON, Canada; j School of Policy Studies, Queen's University, Kingston, ON, Canada; k Division of Clinical and Translational Research, Department of Anesthesiology, Washington University Pain Center, Washington University School of Medicine, St Louis, MO; l Department of Anesthesiology, Perioperative, and Pain Medicine, and Pediatrics, Stanford University School of Medicine, Stanford, Palo Alto, CA; m Department of Surgery and Cancer, Faculty of Medicine, Imperial College London, London, United Kingdom; n Department of Anesthesia, University of California, San Francisco, CA; o Sutter Health, CPMC Research Institute, California Pacific Medical Center Research Institute, San Francisco, CA; p Division of Pain Medicine, Department of Anesthesiology, University of California San Diego, San Diego, CA; q Pain Research, Nuffield Department of Clinical Neurosciences, The Churchill, University of Oxford, Oxford, United Kingdom

**Keywords:** Cannabinoids, Cannabis, Meta-analysis, Pain, Protocol, Systematic review, Overview

## Abstract

Pain is an experience that affects many people worldwide and is associated with higher mortality and lower quality of life. Cannabinoid, cannabis, and cannabis-based medicines (CBMs) are thought to reduce pain, but a proliferation of different products has led to variability in trials, creating a challenge when determining the assessment of efficacy in systematic reviews. We will conduct 2 systematic reviews commissioned by the International Association for the Study of Pain Task Force on the use of cannabinoids, cannabis, and CBMs for pain management: first, an overview review of systematic reviews to summarise the evidence base and second, a systematic review of randomised controlled trials of cannabinoids, cannabis, and CBMs. In these reviews we will determine the harm and benefit of CBM from the current literature and will interpret the findings in light of the quality of evidence and reviews included. We will search online databases and registries in any language for systematic reviews and randomised controlled trials. We will include studies that evaluate any cannabinoid or CBM vs any control for people with acute and chronic pain. Our primary outcomes for both reviews are the number of participants achieving (1) a 30% and (2) 50% reduction in pain intensity, (3) moderate improvement, and (4) substantial improvement. A number of secondary outcome measures will also be included. We will assess risk of bias and quality of evidence. We will analyse data using fixed and random effect models, with separate comparators for cannabis and CBMs. Prospero ID (CRD42019124710; CRD42019124714).

## Abstract 2

Supplemental Digital Content is Available in the Text.

## 1. Introduction

In 2018, the International Association for the Study of Pain established a Task Force on the use of cannabis and cannabinoid-based medicinal products for pain management, broadly conceptualised. It has 4 Work Packages focused on (1) basic science, including medicinal chemistry, compound classification, pharmacology, and assessment of efficacy in preclinical studies, (2) evidence synthesis on clinical efficacy and where possible effectiveness, (3) evidence synthesis on potential harms—going beyond adverse events reported in randomized controlled clinical trials to include harms reported in other study designs,^12,23^ and (4) the societal impact including changing policy and political practice. This review is part of the second Work Packages and is focused on summarizing the evidence for both the efficacy and adverse events as measured primarily within individual randomized controlled trials (RCTs) of cannabinoids, cannabis, and cannabis-based medicines (CBMs).

Pain is a common symptom of a wide variety of common conditions and the primary reason most patients seek health care.^21^ Globally, tension-type headache is the primary cause of morbidity, with musculoskeletal and neuropathic pain also common.^32^ The incidence of chronic pain is routinely estimated to be between 11% and 40% of the population, with as many as 10% reporting severe pain.^4,11^ Chronic pain has a larger impact on quality of life than other common chronic conditions,^40^ and there is a graded increase in mortality as pain severity increases in older adults, especially for patients who report walking disability.^38,39^

Pharmacological treatment of pain can provide considerable improvements, including reduced pain intensity and increased function. However, this benefit is only reported by a minority of patients,^26^ with the exception of patients reporting acute pain after surgery and cancer pain.^27^ For most other patients reporting primarily chronic pain, most pharmacological interventions do not improve quality of life, meaningfully reduce pain intensity, or improve physical functioning.^26^ These findings all relate to adult data. For children and adolescents, there are little data of any kind to guide practice.^8,9^

Cannabis plant material typically contains over 450 different compounds, with over 100 classified as phytocannabinoids. The 2 phytocannabinoids that have been most studied to date in the context of medical research are delta 9-tetrahydrocannabinol (THC, the main psychoactive constituent) and cannabidiol (CBD). Preclinical data support an influence of cannabinoids and modulators of the body's own endogenous cannabinoids (endocannabinoids) on nociception.^30,41,48^ The analgesic effects of THC are mediated primarily through agonism of cannabinoid_1_ (CB_1_) and cannabinoid_2_ (CB_2_ receptors), with the former being chiefly responsible for its psychoactive effects. By contrast, CBD does not activate CB_1_ or CB_2_ receptors and appears to have a complex pharmacology with activity at a number of different targets that include, but are not limited to: 5-HT_1A_ receptor agonism, negative allosteric modulation of CB_1_, GPR55 antagonism, TRPV1 activation, PPARγ activation, and reuptake inhibition (eg, anandamide and adenosine).^3,18,20,31,34,35,43^

The content of THC and CBD in medical cannabis is highly variable, with typical ranges from 1% to 22% THC and 0.05% to 9% CBD. By contrast, the THC/CBD concentration in THC/CBD oromucosal spray (nabiximols) and the THC content in plant-derived and synthetic THC are standardised. There is considerable research interest in the use of medicinal cannabis and CBMs, including for pain. As such, a high-quality review of the literature is needed at this time to assess the quality of evidence and to provide recommendations to clinicians and policymakers on how beneficial cannabinoids, cannabis, and CBM are for people with pain, and what adverse events are associated with them. Table 1 (Ref. 15) provides a summary of current terminology, definitions, and typical products.

**Table 1:**
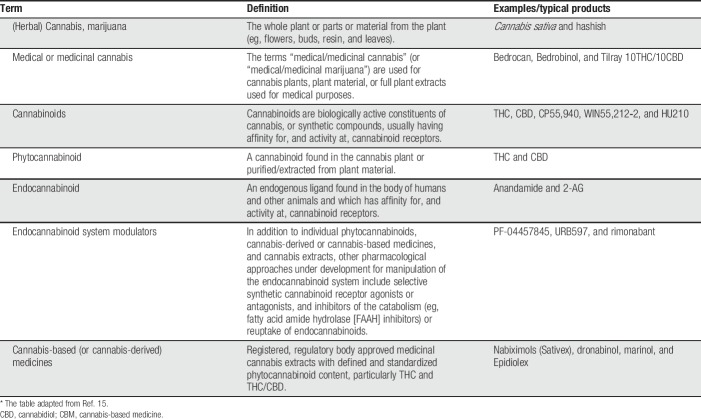
Description of types of CBMs.*

## 2. Aims and objectives

In this protocol, we outline 2 systematic reviews. First, we aim to conduct an overview review of systematic reviews and second, to conduct a systematic review of RCTs. These 2 reviews will be individually published.

The aim of the overview review is to provide a comprehensive summary of the evidence, including an estimate of the therapeutic efficacy and summary of the adverse events of cannabinoid and CBM for pain management. Our goal is to review all published systematic reviews that have attempted to summarise the evidence for efficacy and adverse events reported in systematic reviews evaluating the evidence from RCTs. We recognise the existence of multiple reviews of the primary literature, and we note that different reviews have produced different conclusions. Our aim is to provide an authoritative overview of these reviews and help in understanding the variability across reviews with a focus on their quality. We will assess each review's quality, scope, and reported result against the highest reporting standards.

The aim of the systematic review of RCTs is to provide a comprehensive summary of the evidence from primary RCTs of cannabinoids, cannabis, and CBMs in clinical acute and chronic pain management, across the lifespan. We will (1) provide estimates of the efficacy and adverse events from trial data and (2) provide an assessment of the risk of bias and quality of evidence.

## 3. Methods

### 3.1. Protocol registration

The protocols for these 2 systematic reviews are registered on Prospero (overview of systematic reviews Prospero ID CRD42019124710; systematic review for RCTs Prospero ID CRD42019124714). We have followed the Preferred Reporting Items for Systematic Review and Meta-Analysis Protocols.^22^ This protocol has used the Cochrane Pain, Palliative, and Supportive Care review group template, which are used in a number of other Cochrane Reviews (including, but not limited to Refs. 25 and 47).

### 3.2. Type of participants

Across both reviews, we will include people with acute or chronic pain. Chronic pain is defined as continuous or recurrent pain lasting for longer than 3 months. Acute or chronic pain includes, but is not limited to, the following conditions: abdominal pain, cancer pain, headache, migraine, acute or chronic neuropathic pain, acute or chronic musculoskeletal pain, pelvic pain, menstrual pain, acute postoperative pain, or any other form of pain. We will include people with pain across the lifespan (including children) and reviews of trials conducted in any setting, in any part of the world. However, we will exclude trials of people undergoing experimental pain procedures. For the review of RCTs only, trials must include 30 participants/arm post-treatment. Trials that include smaller sample sizes are more likely to produce larger effects.^6,42^

### 3.3. Types of interventions and comparators

We will include any type of cannabinoid product, natural or synthetic, delivered by any route of administration in both reviews. We will include any control, including placebo or active pain therapy, pharmacological or nonpharmacological. Trials that deliver cannabinoids, cannabis, or CBMs in addition to other drugs will also be included. We will only include systematic reviews and trials that have the intention of decreasing pain intensity in participants.

### 3.4. Types of outcomes

The primary and secondary outcomes will be as follows for both reviews:

#### 3.4.1. Primary outcomes

We will extract the following 4 primary outcomes:

(1) The proportion of people with at least 30% pain intensity reduction;
(2) The proportion of people with at least 50% pain intensity reduction;
(3) Moderate improvement defined by IMMPACT^7^; and
(4) Substantial improvement defined by IMMPACT.^7^

#### 3.4.2. Secondary outcomes

We will extract the following secondary outcomes for trials, where available:

(1) Continuous assessments of pain intensity (eg, using a numerical rating scale or visual analogue scale);
(2) The proportion of people who decreased pain from moderate/severe to mild;
(3) Disability or physical functioning measures;
(4) Emotional functioning (eg, anxiety and depression);
(5) Carer Global Impression of Change;
(6) Quality of life as defined by validated scales;
(7) The number of adverse events: Adverse events will include measures of harm, including withdrawal due to serious adverse events, withdrawal due to adverse events, patients reporting any adverse event, and particular adverse events (especially central nervous system and cardiovascular adverse events). Following the PRISMA Harms Checklist, we will describe how adverse events were addressed, how they were reported, and over what time period the harm was experienced^49^;
(8) Requirement for rescue analgesia;
(9) Sleep duration and quality; and
(10) Onset and duration of analgesic effects (when relevant in acute pain trials).

### 3.5. Search method and study selection

For the overview review, we will search PubMed, EMBASE, DARE, and the Cochrane Controlled Register of Trials (CENTRAL) for systematic reviews of cannabinoids, cannabis, and CBMs for people with pain. For the review of RCTs, we will search PubMed, EMBASE, and CENTRAL (see Appendix 1 for search strategies, available at http://links.lww.com/PR9/A42).

For both reviews, 2 authors will independently sift the titles and abstracts identified in the database search. A third author will resolve any disagreements. We will include any peer-reviewed publication that investigates the therapeutic effects of any cannabinoid preparation, given by any route of administration, for relief of pain, compared with placebo or a different active treatment. We will not include trials based on the measures they report. We will not seek the unpublished literature or conference abstracts. However, we will search online trial registry databases including clinicaltrials.gov, EudraCT, and Prospero where appropriate. We will not restrict the searches on language or date. We will conduct reference and citation searches of included reviews and trials.

For the systematic review of RCTs, where possible, we will match included RCTs to their trial registry identifier. We will include trials registered but yet to report as unpublished in “on-going studies and awaiting classification.” These studies will not be included in the analyses and will be described separately to the included studies. We will assess and, if possible, describe any reasons for nonpublication.

### 3.6. Data extraction

For both reviews, 2 authors will independently extract data from included trials. A third author will resolve any disagreements. We will extract the following data from each study:

(1) Review/study characteristics, eg, design, participants enrolled, age, sex, pain condition, enrolment method, and inclusion/exclusion criteria.
(2) Intervention and comparator characteristics, eg, type of cannabinoid, dose, route of administration, and comparator.
(3) Outcomes—we will extract any outcomes listed in the primary and secondary outcomes of this review. We will extract outcomes at short term (between up to 7 days after administration) and long term (greater than or equal to 7 days after administration).

### 3.7. Validity assessment

For the overview review, we will conduct additional validity checks. For reviews and results of reviews in pain to be valid, a number of other criteria will be taken into account, and we will also evaluate these points in the assessment of the systematic reviews. This will be done in duplicate, and disagreements will be discussed and arbitrated by a third author. These include the following:

(1) Did the review use a defined diagnostic criterion for pain conditions?
(2) Did the reviews include only studies in which patients made their own assessment of pain? This is important because research has found that professionals disagree with patient assessments and often significantly underestimate pain.^36^
(3) Did the reviews use studies with defined minimum pain intensity? It is usual to define a minimal pain intensity of moderate or severe pain, as only mild pain can be acceptable to patients, and reduce the sensitivity of a trial to demonstrate an analgesic effect from an intervention.
(4) Did the reviews examine study size as a confounding factor in any analysis of efficacy? There is increasing evidence of the importance of small trial size, both because of random chance,^2,28,44^ and as an important source of bias.^5,6,10,19,29^ Systematic reviews have been criticised for being overconfident of results with inadequate data.^1,33,46^
(5) Did the review examine susceptibility to publication bias? Statistical tests for the presence of publication bias have been shown to be unhelpful.^45^ For each review with dichotomous numerical data, we will assess the possible effects of publication bias by calculating the number of participants in studies with zero effect (relative risk of 1, or risk difference of zero) needed to give a number needed to treat (NNT) too high to be clinically relevant.^24^ In this case, we will use as a cutoff for clinical relevance, an NNT of 10 or above.

### 3.8. Risk of bias

For the systematic review of RCTs, 2 authors will independently assess the risk of bias of included studies using the Cochrane risk of bias tool,^16^ and a third author will resolve disagreements. We will assess the following risk of bias categories, making judgements using the following criteria. This section uses suggested wording from the Cochrane Pain, Palliative, and Supportive Care review group template, which are used in a number of other Cochrane Reviews (including, but not limited to Refs. 25 and 47).

(1) Random sequence generation (checking for possible selection bias): We will assess the method used to generate the allocation sequence as low risk of bias (any truly random process, eg, random number table; computer random number generator); unclear risk of bias (insufficient detail about the method of randomisation to be able to judge the generation as “low” or “high” risk of bias). Studies using a nonrandom process (eg, odd or even date of birth; hospital or clinic record number) will be excluded.
(2) Allocation concealment (checking for possible selection bias): The method used to conceal allocation to interventions before assignment determines whether intervention allocation could have been foreseen in advance of, or during recruitment, or changed after assignment. We will assess the methods as low risk of bias (eg, telephone or central randomisation; consecutively numbered sealed opaque envelopes); unclear risk of bias (insufficient detail about the method of randomisation to be able to judge the generation as “low” or “high” risk of bias). Studies that do not conceal allocation (eg, open list) will be excluded.
(3) Blinding of participants and personnel (checking for possible performance bias): We will assess the methods used to blind study participants and personnel from knowledge of which intervention a participant received. We will assess the methods as low risk of bias (no blinding or incomplete blinding, but the review authors judge that the outcome was not likely to be influenced by lack of blinding, or blinding of participants and key study personnel ensured, and unlikely that the blinding could have been broken); unclear risk of bias (insufficient detail about the method of randomisation to be able to judge the generation as “low” or “high” risk of bias, or the study does not address this outcome); or high risk of bias (no blinding or incomplete blinding, and the outcome was likely to be influenced by lack of blinding, or blinding of key study participants and personnel attempted, but likely that the blinding could have been broken, and the outcome was likely to be influenced by lack of blinding).
(4) Blinding of outcome assessment (checking for possible detection bias): We will assess the methods used to blind study participants and outcome assessors from knowledge of which intervention a participant received. We will assess the methods as low risk of bias (no blinding of outcome assessment, but the review authors judge that the outcome measurement was not likely to be influenced by lack of blinding, or blinding of outcome assessment ensured, and unlikely that the blinding could have been broken); unclear risk of bias (insufficient detail about the method of blinding to be able to judge the generation as “low” or “high” risk of bias, or the study does not address this); high risk of bias (no blinding of outcome assessment, and the outcome measurement was likely to be influenced by lack of blinding, or blinding of outcome assessment, but likely that the blinding could have been broken, and the outcome measurement was likely to be influenced by lack of blinding).
(5) Incomplete outcome data (checking for possible attrition bias due to the amount, nature, and handling of incomplete outcome data): We will assess the methods used to deal with incomplete data as low risk (no missing outcome data; reasons for missing outcome data unlikely to be related to true outcome (for survival data, censoring unlikely to be introducing bias); missing outcome data balanced in numbers across intervention groups, with similar reasons for missing data across groups; missing data have been imputed using “baseline observation carried forward” analysis); unclear risk of bias (insufficient reporting of attrition/exclusions to permit a judgement of “low risk” or “high risk” [eg, number randomised not stated, no reasons for missing data provided, or the study did not address this outcome]); high risk of bias (reason for missing outcome data is likely to be related to true outcome, with either imbalance in numbers or reasons for missing data across intervention groups; “as-treated” analysis performed with substantial departure of the intervention received from that assigned at randomisation; potentially inappropriate application of simple imputation).
(6) Selective reporting (checking for reporting bias): We will assess reporting biases because of selective outcome reporting. We will judge studies as low risk of bias (the study protocol is available and all of the study's prespecified [primary and secondary] outcomes that are of interest in the review have been reported in the prespecified way; the study protocol is not available, but it is clear that the published reports include all expected outcomes, including those that were prespecified [convincing text of this nature may be uncommon]); unclear risk of bias (insufficient information available to permit a judgement of “low risk” or “high risk”); high risk of bias (not all of the study's prespecified primary outcomes have been reported; one or more primary outcomes have been reported using measurements, analysis methods, or subsets of the data (eg, subscales) that were not prespecified; one or more reported primary outcomes were not prespecified (unless clear justification for their reporting is provided, such as an unexpected adverse effect); one or more outcomes of interest in the review have been reported incompletely so that they cannot be entered in a meta-analysis; the study report failed to include results for a key outcome that would be expected to have been reported for such a study).

### 3.9. Assessment of methodological quality of included reviews

For the overview review, we will assess each included review using AMSTAR-2, which determines rigorous methodological review quality.^37^ Two authors will assess each review using the criteria, and a third author will resolve disagreements. We will also assess how each review compares with Cochrane assessments of risk of bias,^16^ together with other methodological issues, such as imputation bias, or small size effects.

### 3.10. Quality of the evidence (GRADE)

This section uses suggested wording from the Cochrane Pain, Palliative, and Supportive Care review group template, which are used in a number of other Cochrane Reviews (including, but not limited to Refs. 25 and 47).

For both reviews, we will use GRADE to assess the quality of the evidence in each review. Two review authors will rate the quality of each outcome. The GRADE approach uses 5 considerations (study limitations, unexplained heterogeneity and inconsistency of effect, imprecision, indirectness, and publication bias) to assess the quality of the body of evidence for each outcome. The GRADE system uses the following criteria for assigning the grade of evidence:

(1) High: we are very confident that the true effect lies close to that of the estimate of the effect;
(2) Moderate: we are moderately confident in the effect estimate; the true effect is likely to be close to the estimate of effect, but there is a possibility that it is substantially different;
(3) Low: our confidence in the effect estimate is limited; the true effect may be substantially different from the estimate of the effect;
(4) Very low: we have very little confidence in the effect estimate; the true effect is likely to be substantially different from the estimate of effect.

Factors that may decrease the quality level of a body of evidence are as follows:

(1) Limitations in the design and implementation of available studies suggesting high likelihood of bias;
(2) Indirectness of evidence (indirect population, intervention, control, and outcomes);
(3) Unexplained heterogeneity or inconsistency of results (including problems with subgroup analyses);
(4) Imprecision of results (wide confidence intervals); and
(5) High probability of publication bias.

We will decrease the grade rating by one (−1) level (from high to moderate quality of evidence), 2 (−2) levels (to low-quality evidence), or 3 (−3) levels (to very low quality of evidence). Outcomes can be downgraded a maximum of 3 levels using the following criteria:

(1) Serious (−1) or very serious (−2) study limitations.
(2) Some (−1) or considerable (−2) inconsistency of results.
(3) Some (−1) or considerable (−2) uncertainty about directness.
(4) Some (−1) or considerable (−2) imprecision.
(5) Some (−1) or considerable (−2) probability of reporting bias.

There may be circumstances where the overall rating for a particular outcome needs to be adjusted as recommended by GRADE guidelines.^14^ Examples might be where there are so few participants that the results are highly susceptible to the random play of chance, or if studies use last observation carried forward imputation in circumstances where there are substantial differences in adverse event withdrawals. In circumstances such as this, there would be little confidence in the result, which would be downgraded 3 levels, to very low quality. In circumstances where there are no data reported, we will report the level of evidence as very low quality.^13^

### 3.11. “Summary of findings” tables

For both reviews, we will produce 2 main “summary of findings” tables; cannabis vs control, and CBMs (to include individual cannabinoids) vs control. Furthermore, we will rate the quality for all subgroup analyses conducted and describe reasons for downgrading. We will include the following 7 outcomes: 50% pain reduction, 30% pain reduction, moderate improvement, substantial improvement, adverse events, physical functioning, and emotional functioning.

### 3.12. Data synthesis

For the overview review, we will evaluate the strengths and weaknesses of systematic reviews of cannabinoids in treating pain of any source. We plan to show this in a tabular form. We will only analyse evidence that falls into the EPOC groups 4 and 5 (Table 2). Where enough data are available, we will combine data from systematic reviews into a meta-analysis to investigate our primary or secondary outcomes.

**Table 2:**
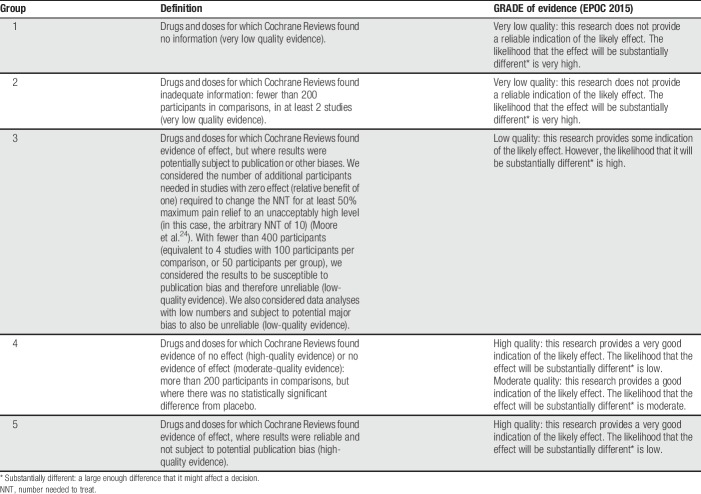
Definitions of hierarchy of groups and EPOC criteria.

For the review of RCTs, we will combine data in meta-analyses where possible. Both reviews will conduct the same analyses and subgroup analyses where data are available.

We will use standardised mean differences for continuous outcomes and risk ratios for dichotomous outcomes. We will calculate the NNT to benefit and NNT to harm where appropriate. Heterogeneity will be interpreted following the Cochrane Handbook.^17^ Adverse events will be entered into meta-analyses and calculated using risk ratios and 95% confidence intervals. Where zero events occur in trials, we will enter 0.5 into each cell of the analysis.^49^ Where possible, we will describe any assessment of possible causality of adverse events.

We will conduct comparisons of cannabis vs control, and CBMs (to include individual cannabinoids) vs control, for each of our named outcomes to determine efficacy. We will conduct the following 4 primary analyses, which will include all trials, conducted with a subgroup analysis of low risk of bias vs unclear/high risk of bias trials, at 2 time points:

(1) Cannabis vs control at short-term follow-up (between up to 7 days after administration);
(2) Cannabis vs control at long-term follow-up (greater than or equal to 7 days after administration);
(3) Cannabis-based medicine vs control at short-term follow-up (between up to 7 days after administration); and
(4) Cannabis-based medicine vs control at long-term follow-up (greater than or equal to 7 days after administration).

We will conduct sensitivity analyses where appropriate to investigate the impact of risk of bias and study quality.

#### 3.12.1. Subgroup analyses

In addition, where enough data are available, we will conduct the following subgroup analyses at 2 time points outlined above:

(1) Age of participants (2–10 years, 11–17 years, 18–64 years, and older than 65 years);
(2) The drug subtype under investigation for cannabis and CBMs;
(3) Type of comparator;
(4) Route of administration;
(5) Dose of treatment;
(6) Type of pain experienced (acute, neuropathic pain, fibromyalgia, musculoskeletal pain, headache/migraine, etc.); and
(7) Cannabis or CBM administered adjunctively vs nonadjunctively to other medicines.

## 4. Discussion

This overview review and review of RCTs forms part of a wider programme of work requested by the International Association for the Study of Pain (www.iasp-pain.org) to examine the role of cannabinoid, cannabis, and CBMs in the treatment of pain. It is intended that the 2 products can be used to help in future study design and reporting, to ensure the efficient and appropriate design, conduct, and analysis of clinical trials to the benefit of people with pain. We will provide recommendations based on evidence-based findings from these reviews on the clinical use of cannabis for people with pain.

## Disclosures

L. Degenhardt reports grants from Indivior and grants from Seqirus, outside the submitted work. D.P. Finn reports grants from Randox Ltd, grants from Alkermes, Inc, and grants from Shionogi Ltd, outside the submitted work. N.B. Finnerup reports personal fees from Grünenthal, personal fees from Teva Pharmaceuticals, personal fees from Astellas, personal fees from Novartis Pharma, personal fees from Mitsubishi Tanabe Pharma, personal fees from Merck, grants from PainCare, IMI2 (Innovative medicines initiative), which is a EU public–private consortium with a grant provided by the EU and the companies involved are: Grunenthal, Bayer, Eli Lilly, Esteve, Novartis, and Teva, outside the submitted work. I. Gilron reports personal fees from Adynxx, personal fees from Biogen, personal fees from Eupraxia, personal fees from Novaremed, personal fees from Teva, nonfinancial support from Canopy Health, nonfinancial support from Toronto Poly Clinic, and nonfinancial support from CannTrust, outside the submitted work. S. Haroutounian reports grants from Pfizer Inc and personal fees from Medoc Ltd, outside the submitted work. A.S.C. Rice is part of the Presidential Task Force of the IASP, during the conduct of the study; personal fees from Imperial College Consultants, other from Spinidex/Novartis, outside the submitted work. In addition, A. Rice has a patent: Rice A.S.C, Vandevoorde S., and Lambert D. M Methods using N-(2propenyl)hexadecanamide and related amides to relieve pain. WO2005/079771 pending, and a patent Okuse K. et al. Methods of treating pain by inhibition of vgf activity EP13702262.0/WO2013 110945 pending. M. Wallace reports personal fees from Insys, outside the submitted work. R.A. Moore reports personal fees from Novartis and personal fees from RB, outside the submitted work. The remaining authors have no conflicts of interest to declare.

Cochrane Review Group funding acknowledgement: this project was partly supported by the National Institute for Health Research, via Cochrane Infrastructure funding to the Cochrane Pain, Palliative, and Supportive Care Review Group (PaPaS). The views and opinions expressed therein are those of the authors and do not necessarily reflect those of the Systematic Reviews Programme, NIHR, NHS, or the Department of Health.

## Appendix A. Supplemental digital content

Supplemental digital content associated with this article can be found online at http://links.lww.com/PR9/A42.
